# TFEB regulates dendritic cell antigen presentation to modulate immune balance in asthma

**DOI:** 10.1186/s12931-024-02806-1

**Published:** 2024-04-25

**Authors:** JinYing Xiang, Bo Liu, Yan Li, Yinying Ren, Yuehan Li, Mi Zhou, Jinyue Yu, Zhengxiu Luo, Enmei Liu, Zhou Fu, Fengxia Ding

**Affiliations:** 1 Department of Respiratory Medicine, Ministry of Education Key Laboratory of Child Development and Disorders, Chongqing Engineering Research Center of Stem Cell Therapy, National Clinical Research Center for Child Health and Disorders, China International Science and Technology Cooperation base of Child development and Critical Disorders, Children’s Hospital of Chongqing Medical University, No. 136, Zhongshan 2nd Road, Yuzhong Dis, 400014 Chongqing, PR China; 2 Department of Cardiothoracic Surgery, Ministry of Education Key Laboratory of Child Development and Disorders, Chongqing Engineering Research Center of Stem Cell Therapy, National Clinical Research Center for Child Health and Disorders, China International Science and Technology Cooperation base of Child development and Critical Disorders, Children’s Hospital of Chongqing Medical University, No. 136, Zhongshan 2nd Road, Yuzhong Dis, 400014 Chongqing, PR China; 3 Bristol Medical School, University of Bristol, Bristol, UK; 4 Great Ormond Street Institute of Child Health, University College London, London, UK

**Keywords:** TFEB, Dendritic cell, Asthma, Antigen presentation, T cell

## Abstract

**Objective:**

Asthma stands as one of the most prevalent chronic respiratory conditions in children, with its pathogenesis tied to the actived antigen presentation by dendritic cells (DCs) and the imbalance within T cell subgroups. This study seeks to investigate the role of the transcription factor EB (TFEB) in modulating the antigen presentation process of DCs and its impact on the differentiation of T cell subgroups.

**Methods:**

Bone marrow dendritic cells (BMDCs) were activated using house dust mites (HDM) and underwent RNA sequencing (RNA-seq) to pinpoint differentially expressed genes. TFEB mRNA expression levels were assessed in the peripheral blood mononuclear cells (PBMCs) of both healthy children and those diagnosed with asthma. In an asthma mouse model induced by HDM, the TFEB expression in lung tissue DCs was evaluated. Further experiments involved LV-shTFEB BMDCs co-cultured with T cells to explore the influence of TFEB on DCs’ antigen presentation, T cell subset differentiation, and cytokine production.

**Results:**

Transcriptomic sequencing identified TFEB as a significantly differentially expressed gene associated with immune system pathways and antigen presentation. Notably, TFEB expression showed a significant increase in the PBMCs of children diagnosed with asthma compared to healthy counterparts. Moreover, TFEB exhibited heightened expression in lung tissue DCs of HDM-induced asthmatic mice and HDM-stimulated BMDCs. Silencing TFEB resulted in the downregulation of MHC II, CD80, CD86, and CD40 on DCs. This action reinstated the equilibrium among Th1/Th2 and Th17/Treg cell subgroups, suppressed the expression of pro-inflammatory cytokines like IL-4, IL-5, IL-13, and IL-17, while augmenting the expression of the anti-inflammatory cytokine IL-10.

**Conclusion:**

TFEB might have a vital role in asthma’s development by impacting the antigen presentation of DCs, regulating T cell subgroup differentiation, and influencing cytokine secretion. Its involvement could be pivotal in rebalancing the immune system in asthma. These research findings could potentially unveil novel therapeutic avenues for treating asthma.

**Supplementary Information:**

The online version contains supplementary material available at 10.1186/s12931-024-02806-1.

## Introduction

Asthma is among the prevalent chronic respiratory conditions in children, characterized by chronic airway inflammation, hyperresponsiveness, and irreversible airway wall remodeling [1]. Allergic asthma, the most prevalent type of the disease, results from exposure to innocuous environmental allergens such as house dust mites (HDM), pollen, and fungi [2]. The development of asthma is directly linked to the disruption of the body’s immune homeostasis, in which the activation of antigen-presenting cells and the imbalance between effector T cells and regulatory T cells (Treg) play a crucial role in this process [3]. DCs are the most effective antigen-presenting cells in stimulating adaptive immune responses. Upon stimulation by external triggers, these cells’ antigen presentation functions are activated, thereby promoting the immune responses mediated by effector T cells [4]. DCs are also the most common antigen-presenting cells in the lungs, playing a critical role in maintaining pulmonary immune homeostasis [5, 6]. When the respiratory tract is exposed to an allergen, DCs process it into small peptides and migrate to the mediastinal lymph nodes, binding to the MHC II receptor and co-stimulatory molecules on the surface of naïve CD4 + T helper cells, consequently achieving antigen presentation of the allergen. Subsequently, naïve CD4 + T helper cells differentiate into various T cell subtypes, producing corresponding cytokines [7, 8].

TFEB is a member of the MiTF/TFE family of basic helix-loop-helix leucine zipper transcription factors, linked to a range of conditions including neurodegenerative diseases, metabolic disorders, obesity, tumors, and lysosomal storage diseases [9, 10]. Previous research has shown that TFEB is involved in both the non-specific and adaptive immune responses [11]. When macrophages are exposed to pathogens, TFEB facilitates the transcription of various chemokines and pro-inflammatory cytokines that are essential for the immune response [12]. Additionally, TFEB’s transcriptional activity is upregulated during CD4 + T cell activation and significantly contributes to humoral immunity by enhancing the production of IgG and IgA [13]. Recent studies indicate that TFEB aids DCs in capturing and transporting antigens to lysosomes. TFEB elevates intracellular Ca2 + concentration by activating the lysosomal calcium channel TRPML1, which drives the fusion of lysosomes with the plasma membrane. This process facilitates the transportation of antigen-bound MHC II molecules for expression on the outer surface of the cell membrane, resulting in a shift towards MHC II for cross-antigen presentation, and enhancing the activation of CD4 + T cells [14, 15]. Additionally, the migration of DCs from the periphery to the lymph nodes is regulated by a TFEB-TRPML1 axis feedback loop, which originates from lysosomes [16]. These findings suggest that TFEB could play a pivotal role as a transcription factor in initiating antigen presentation by DCs and mediating the immune response of CD4 + T cells. However, it is currently unclear how TFEB changes in asthma and what impact it has on the antigen-presenting function of DCs and T-cell differentiation. Therefore, in this study, we employed RNA-seq to identify differentially expressed genes, while concurrently evaluating the expression of TFEB mRNA in peripheral blood mononuclear cells (PBMCs) from both healthy children and children diagnosed with asthma. Moreover, by constructing a mouse model of HDM-induced asthma and a co-culture system of bone marrow dendritic cells (BMDCs) and T cells, we comprehensively analyze the role of TFEB in the process of antigen presentation by DCs and in mediating the immune response of CD4 + T cells.

## Materials and methods

### Cell culture

BMDCs were obtained from mouse femurs and tibiae according to previously described methods [17]. T cells were obtained from the spleens of C57BL/6 mice using the Mouse Spleen Lymphocyte Isolation Kit (Hao Yang Bio, Tianjin, China) according to the manufacturer’s instructions. T cells were inoculated with BMDCs in a 5:1 ratio into 12-well plates and co-cultured for 36 h with or without HDM (20ug/ml) stimulation.The co-cultured cell culture supernatant and co-cultured cells were collected for subsequent experiments. All cells were cultured in an incubator at 37 °C containing 5% CO2.

### RNA-Seq and data analysis

BMDCs with stable culture condition were inoculated into 6 cm petri dishes, and 5 petri dishes were randomly selected as experimental group to be stimulated with HDM (20ug/ml), and 5 other petri dishes were randomly selected as control group to be not stimulated with HDM. After incubating at 37 °C with 5% CO2 for 36 h, collected control and experimental groups for RNA-seq (Boho Biotech Co., Ltd, Shanghai, China). Differential genes (DEGs) between the two groups were screened according to the criteria of |log2FC|>1 and *P*<0.05, and the volcano map was plotted using the “ggplot2” R package, and the enrichment analysis was performed by the “clusterProfiler” R package.

### Human subjects

Thirty-eight subjects were recruited from the outpatient department of the Children’s Hospital of Chongqing Medical University, comprising a ratio of approximately 1:1.5 between healthy children (*n* = 15) and children diagnosed with asthma (*n* = 23). Inclusion criteria for the asthma group included children aged 4 years or older who met the diagnostic criteria for asthma outlined in the Global Initiative for Asthma (GINA) Management and Prevention guidelines published in 2023 [18]. Patients with other respiratory diseases, a history of respiratory infections within the last 2 weeks, and a history of immunosuppressant or glucocorticoid use within 4 weeks before the start of the study were excluded. Healthy children over 4 years of age without respiratory disease and no recent history of infection were used as controls. A total of 3 ml of peripheral blood was collected from each subject, from which peripheral blood mononuclear cells (PBMCs) were isolated using the Human Peripheral Mononuclear Cells Isolation Kit (Hao Yang Bio, Tianjin, China). Total RNA was then extracted from the PBMCs using the SimplyP Total RNA Extraction Kit (BioFlux, USA), following the manufacturer’s instructions.

### HDM-induced allergic asthma mouse model

C57BL/6 mice (female, 6–8 weeks old) were purchased from Chongqing Enswell Biotechnology Co Ltd (Chongqing, China). Mice were housed in an environment with a humidity level of 40–70%, at a temperature of 24 °C, and subjected to a 12-hour light/dark cycle. Adequate food and water were provided and all were sterilised. Twenty female C57BL/6 mice were randomly assigned to the control group (*n* = 10) and the asthma group (*n* = 10). On days 0 and 14, mice were intranasally administered 40 µl of HDM (20 µg) solution (Greer, Los Angeles, CA, USA) for sensitization, followed by intranasal administration of 40 µl HDM (20 µg) solution on days 21, 23, 25, 27, and 29 for challenge. The control group received saline instead of the HDM solution. On day 31, all mice were sacrificed for relevant experimental tests and analyses [17].

### Lung histopathological staining and immunohistochemistry

The left lung tissue of mice was fixed with 4% paraformaldehyde, embedded in paraffin, and then sliced into 4 μm thick lung tissue paraffin sections. The inflammation around the bronchi, blood vessels, and pulmonary interstitium was assessed using hematoxylin and eosin (H&E) staining, and graded from 0 to 3. A score of 0 indicated no inflammatory response; 1 indicated inflammatory infiltration around the bronchi, blood vessels, and pulmonary interstitium without thickening; 2 indicated obvious inflammatory infiltration and mild thickening around the bronchi, blood vessels, and pulmonary interstitium; and 3 indicated significant inflammatory infiltration and marked thickening around the bronchi, blood vessels, and pulmonary interstitium. For immunohistochemistry, sections were incubated with primary anti-TFEB antibody (1:150, Proteintech, Wuhan, China) overnight, followed by 30 min of incubation with the secondary antibody. DAB (Zhongshang JinQiao Biotechnology Co., Ltd., Beijing, China) was used for visualization. The mean optical density (MOD) was analyzed using Image J software.

### Immunofluorescence of lung sections and cells

4% paraformaldehyde was utilized to fix 4-µm lung tissue paraffin sections and BMDCs, followed by permeabilization with 0.2% Triton X100 (Solarbio, Beijing, China), and blocking with 5% BSA (Solarbio, Beijing, China). The samples were incubated overnight at 4 °C with rabbit anti-mouse TFEB primary antibody (diluted 1:300, Proteintech, Wuhan, China) and CoraLite Plus 488-conjugated CD11c (diluted 1:200, Proteintech, Wuhan, China). Afterward, the samples were incubated at room temperature in the dark for 1 h with FITC- or cy3-conjugated goat anti-rabbit secondary antibody (diluted 1:200, Proteintech, Wuhan, China). DAPI (Beyotime, Shanghai, China) was used as a nuclear stain and incubated in the dark at room temperature for 15 min. Actin-Tracker Red-555 (diluted 1:100, Beyotime, Shanghai, China) was employed as a cytoskeletal dye and incubated for 1 h at room temperature. After mounting with anti-fluorescence quencher (Beyotime, Shanghai, China), the images were captured under a confocal microscope (Nikon, Tokyo, Japan), and analyzed using ImageJ software.

### Bronchoalveolar lavage and differential cell counting

The left bronchus of mice was ligated, and bronchoalveolar lavage fluid (BALF) was collected using pre-cooled PBS. After centrifugation at 2500 rpm for 20 min at 4 °C, the cells were lysed with erythrocyte lysate, and the total BALF cell count was determined using a cell counter. The centrifuged BALF cell sediment was placed on slides, and the cells were stained with rapid Rhee’s staining solution (Built Technology Co., Ltd., Nanjing, China). Subsequently, at least 200 cells were enumerated under the microscope, and the number and proportion of macrophages, neutrophils, eosinophils, and lymphocytes were recorded separately.

### Enzymelinked immunosorbent assay (ELISA)

Mouse ocular blood was collected and centrifuged at 2500 rpm for 15 min at room temperature, and serum was obtained for the determination of IgE levels. According to the protocol, the mouse IgE ELISA kit (Ruixin Bio, Guangzhou, China) was used to measure the total IgE level in the serum. The supernatant of mouse bronchoalveolar lavage fluid (BALF) was obtained after centrifugation at 4 °C for 20 min, and the expression levels of IL-4, IL-10, IL-17, and IFN-γ were measured using the Mouse IL-4, IL-10, IL-17, and IFN-γ ELISA kit (Ruixin Bio, Guangzhou, China). The medium was collected after co-culturing T cells and BMDCs, then centrifuged at 2500 rpm for 15 min at room temperature, and the levels of IL-4, IFN-γ, and IL-17 in the culture medium supernatant were determined using the Mouse IL-4, IFN-γ, and IL-17 ELISA Kit (Ruixin Bio, Guangzhou, China).

### Measurement of airway hyperresponsiveness (AHR)

After the last HDM stimulation for 24 h, the resistance of the lung to different doses of acetylcholine was measured using a non-invasive lung function meter (EMKA Technologies, Paris, France). Mice were placed in a body plethysmograph, and non-invasive whole-body plethysmography (WBP) was utilized to evaluate bronchial constriction in awake, freely active mice by assessing their respiratory physiological parameters. Baseline measurements were recorded prior to stimulating the mice with the bronchoconstrictor acetyl-β-methylcholine (Sigma, Missouri, USA) at doses of 0, 3.125, 6.25, 12.5, 25, and 50 mg/ml using a nebulization system. The lung resistance at each concentration was calculated.

### RNA interference

The shRNA sequences for mouse TFEB (shTFEB-1-5ʹ--3ʹ, shTFEB-2-5ʹ--3ʹ, shTFEB-3-5ʹ--3ʹ) were synthesized by GeneChem company (Shanghai, China). shGFP was used as a control group. BMDCs were seeded into a 24-well plate, and transduced with shGFP or shTFEB at a virus titer of MOI 75. Additionally, HitransG P infection enhancement solution (GeneChem, Shanghai, China) was added to improve the infection efficiency of the lentivirus. The culture medium was replaced with fresh, virus-free medium after 24 h. GFP expression in BMDCs was observed under a fluorescence microscope 72 h after lentiviral infection. Lentivirus infection efficiency was assessed using flow cytometry. The efficiency of TFEB gene knockdown was determined through RT-qPCR, western blotting, and immunofluorescence detection.

### Wound healing assay

To assess the migration ability of the BMDCs, we performed a scratch wound healing assay. The BMDCs transfected with LV-GFP or LV-shTFEB were cultured in a 6-well plate until reaching 100% confluence. Subsequently, the cells were subjected to scratching with a 200µL pipette tip, followed by two washes with PBS and then incubation in medium containing 1% serum. Microscopic images were taken at 6 and 12 h. ImageJ was employed for image analysis and quantitative measurements.

### Flow cytometry

After collecting cultured cells and blocking with rat serum for 30 min, the following flow cytometry antibodies were used for phenotypic analysis: anti-CD11c-APC (Biolegend, California, USA), anti-MHCII-BV421 (Biolegend, California, USA), anti-CD80-Percp/cy5.5 (Biolegend, California, USA), anti-CD86-PE (Biolegend, California, USA), anti-CD40-PE/Cy7 (Elabscience, Wuhan, China). Data was acquired using FACS Canto II (BD Biosciences, USA) and analyzed using FlowJo software.

### RNA extraction and RT-qPCR

We used the SimplyP Total RNA Extraction Kit (BioFlux, USA) to extract total RNA from PBMCs, mouse lung tissue and BMDCs. The RNA was then reverse-transcribed into cDNA using the Evo M-MLV Reverse Transcription Kit (Aike Rui Biotechnology, Hunan, China). RT-qPCR was performed using the SYBR Green Pro Taq pre-mix kit (Aike Rui Biotechnology, Hunan, China) to detect gene expression. Mouse β-actin and human GAPDH were used as internal controls for mouse samples and human PBMCs samples, respectively. The primer sequences we used were as follows:

Mouse TFEB (forward 5’-GCATCAGAAGGTTCGGGAGTATC-3’, reverse 5’-AGGCGCATAATGTTGTCAATGAC-3), mouse IL-4 (forward 5’-GGAGATGGATGTGCCAAACG-3’, reverse 5’-TGGAAGCCCTACAGACGAG-3’), mouse IL-5 (forward 5’-AGCAATGAGACGATGAGGCTT-3’, reverse 5’-TACCCCCACGGACAGTTTGA-3’), mouse IL-6 (forward 5’-GCCTTCTTGGGACTGATGCT-3’, reverse 5’-GGTCTGTTGGGAGTGGTATCC-3’), mouse IL-10 (forward 5’-CAACATACTGCTAACCGACTCCT-3’, reverse 5’-GCCTGGGGCATCACTTCTAC-3’), mouse IL-12 (forward 5’-GCCAGGGTCATTCCAGTCTC-3’, reverse 5’-TGGTTTGGTCCCGTGTGATG-3’), mouse IL-13 (forward 5’-GTATGGAGTGTGGACCTGGC-3’, reverse 5’-TTTTGGTATCGGGGAGGCTG-3’), mouse IFN-γ (forward 5’-GAGGTCAACAACCCACAGGTC-3’, reverse 5’-TCTTC CCCACCCCGAATCA-3’), mouse TRPML1 (forward 5’-CGGTGTCATTCGCTACCTGA-3’, reverse 5’-CAGCGAGCGGAACTTCACAT-3’), mouse Foxp3 (forward 5’-AGAGCGAGAAGGGAGCAGT-3’, reverse 5’-GCAGGGATTGGAGCACTTGT-3’), mouse GATA3 (forward 5’-CCATTACCACCTATCCGCCC-3’, reverse 5’-TTCACACACTCCCTGCCTTC-3’), mouse RORγt (forward 5’-GCAAAGAAGACCCACACCTCA-3’, reverse 5’-ACATTACACTGCTGGCTGCG-3’), mouse T-bet (forward 5’-GCCTACCAGAACGCAGAGAT-3’, reverse 5’-CCCCCAAGCAGTTGACAGTT-3’), mouse β-actin (forward 5’-GTGCTATGTTGCTCTAGACTTCG-3’, reverse 5’-AGCCACAGGATTCCATACC-3’), human TFEB (forward 5’-ACCTGACCCAGAAGCGAGA-3’, reverse 5’-TGAGGATGGTGCCCTTGTTC-3’), human GAPDH (forward 5’-AATGGGCAGCCGTTAGGAAA-3’, reverse 5’-GCGCCCAATACGACCAAATC-3’).

### Western blotting

The mouse lung tissue and BMDCs were lysed using RIPA buffer (Kaige Biotechnology, Jiangsu, China) containing 1x protease inhibitor, 1x phosphatase inhibitor, and 1x PMSF. After centrifugation at 12,000 rpm for 20 min at 4 °C, the supernatant containing the proteins was collected. The protein concentration was measured using a spectrophotometer (Thermo Fisher Scientific, USA). The proteins were separated by SDS-PAGE gel electrophoresis, then transferred to a PVDF membrane, blocked with 5% BSA at room temperature for 1 h. The primary antibodies rabbit anti-mouse TFEB (diluted 1:2000, Proteintech, Wuhan, China) and β-actin (diluted 1:10000, Zen Bioscience, Chengdu, China) were incubated overnight at 4 °C. The membranes were then incubated with HRP-conjugated goat anti-rabbit IgG (diluted 1:1000, Proteintech, Wuhan, China) at room temperature for 1 h. The chemiluminescence was detected using ECL reagent (Zen Bioscience, Chengdu, China) and visualized with a Bio-Rad imaging system (California, USA). Protein expression was quantified using Image J software.

### Statistical analysis

Graph Pad Prism Version 9.5 (Graph Pad Software Inc.) was used for all statistical analyses. Data are presented as mean ± s.e.m. of at least 3 independent experiments. Statistical comparisons were performed with analyzes of variance (ANOVA) or two-tailed Student’s t-test with paired or unpaired wherever appropriate. A *P* value < 0.05 was considered statistically significant.

## Results

### TFEB appears as an up-regulated gene within the RNA-seq findings of BMDCs

**Fig. 1:**
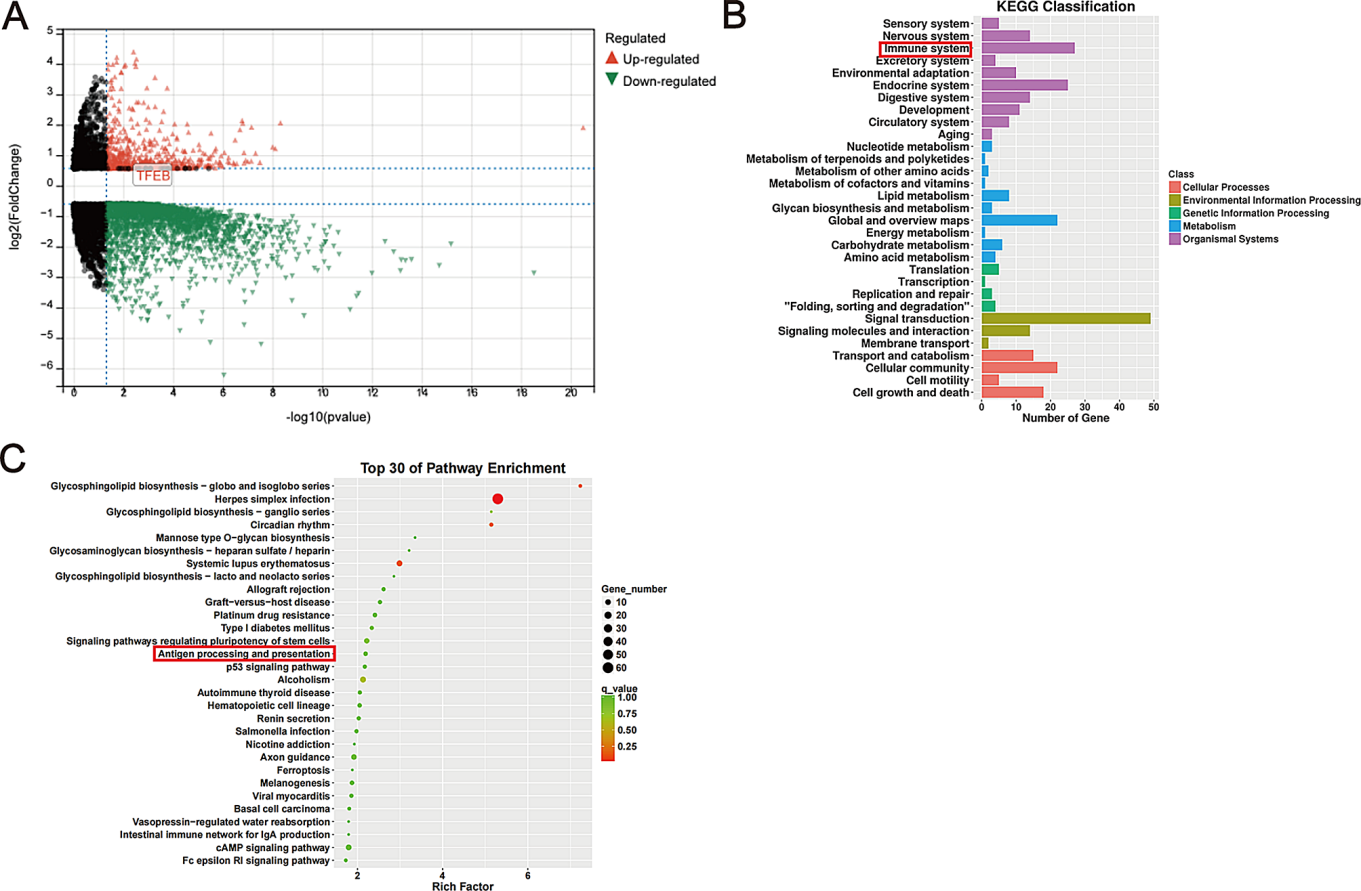
The results of RNA-seq. (**A**) Volcano map of DEGs: Each colored dot represents a DEG based on the criteria of (*P* < 0.01) and |log2FC| > 1; red denotes up-regulation, green denotes down-regulation, and black indicates normally expressed mRNA. (**B**) KEGG classification of DEGs in our sequencing results. (**C**) The 30 most significantly enriched KEGG pathways of DEGs in our sequencing results

In the analysis of the RNA-seq results of BMDCs, we observed that TFEB is a gene that is differentially expressed, with its expression being up-regulated in BMDCs following HDM treatment (Fig. 1A). Additionally, the KEGG Classification results exhibit associations with the immune system (Fig. 1B). Notably, the top 30 enriched pathways identified in the KEGG analysis encompass the antigen processing and presentation pathway (Fig. 1C).

### Characteristics of the subjects

We recruited a total of 38 subjects, comprising 15 healthy children and 23 children diagnosed with asthma (refer to Table 1). The mean serum total IgE levels and allergen-specific IgE levels were notably higher in children with asthma compared to their healthy counterparts (*P* < 0.05). Among children with asthma, 52.17% were allergic to dust mites. Furthermore, a significantly higher percentage of asthmatic children reported a history of secondhand smoke exposure, as well as a family history of allergy or asthma, in contrast to healthy children (*P* < 0.05). Importantly, lung function assessments revealed significantly lower FEV1%pred and FEV1/FVC ratios in children with asthma compared to healthy children, suggesting impaired lung function in the asthmatic group.

**Table 1:** Characteristics of the subjects in the study

	Healthy (*n* = 15)	Asthma (*n* = 23)	p Value
Sex (M/F)	10/5	14/9	0.7261
Age (yrs)	7.36 (0.4140)	6.56 (0.3021)	0.1197
BMI (kg/m²)	17.74 (1.005)	15.68 (0.5245)	0.0548
Serum total IgE level (IU/mL)	73.52 (15.80)	811.3 (167.1)	0.0026
Allergen-specific IgE (kU/L)	0.09556 (0.0632)	30.11 (7.609)	0.0124
Allergens, n(%)	2 (13.33%)	17 (73.91%)	< 0.0001
Dust mite	0 (0.00%)	12 (52.17%)	0.0004
Cat	0 (0.00%)	2 (8.70%)	0.2067
Egg white	1 (6.67%)	1 (4.35%)	0.8679
Others	1 (6.67%)	2 (8.70%)	0.7042
Second-hand smoke, n (%)	6 (40.00%)	17 (73.91%)	0.0373
Familial allergy, n (%)	2 (13.33%)	16 (69.57%)	< 0.0001
Familial asthma, n (%)	0 (0.00%)	8 (34.78%)	0.0031
FEV1 (%pred)	102.71 (2.261)	91.13 (1.998)	0.0006
FVC (%pred)	96.86 (2.369)	97.87 (1.542)	0.7118
FEV1/FVC Age of asthma onset, n (%)	105.89 (1.249)	93.14 (1.866)	< 0.0001

Data presented as mean(SEM) and count (percentage); Definition of abbreviations: BMI = Body mass index, IgE immunoglobulin E, FEV1 = Forced expiratory volume in 1 s, FVC = Forced Vital Capacity; *P* < 0.05 was statistically significant

### Elevated expression of TFEB in PBMCs of children with asthma

**Fig. 2:**
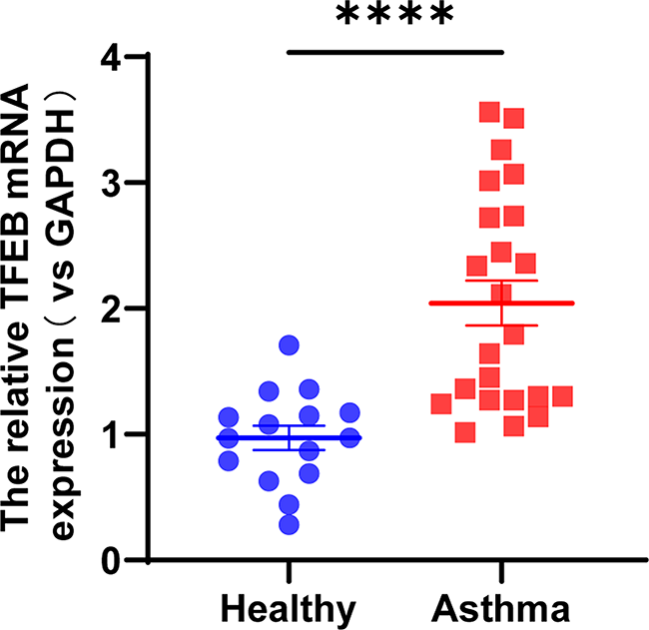
TFEB expression is increased in PBMCs of asthmatic patients. TFEB mRNA expression in PBMCs of healthy subjects (*n* = 15) and asthmatic patients (*n* = 23). ****P < 0.0001

Total RNA was extracted from PBMCs of 38 children, followed by reverse transcription into cDNA, and subsequent analysis using RT-qPCR. The results revealed a significantly elevated expression of TFEB mRNA in the PBMCs of children with asthma compared to healthy controls, demonstrating statistical significance (Fig. 2, *P* < 0.0001).

### Exacerbation of pulmonary inflammation in HDM-induced asthmatic mice

**Fig. 3:**
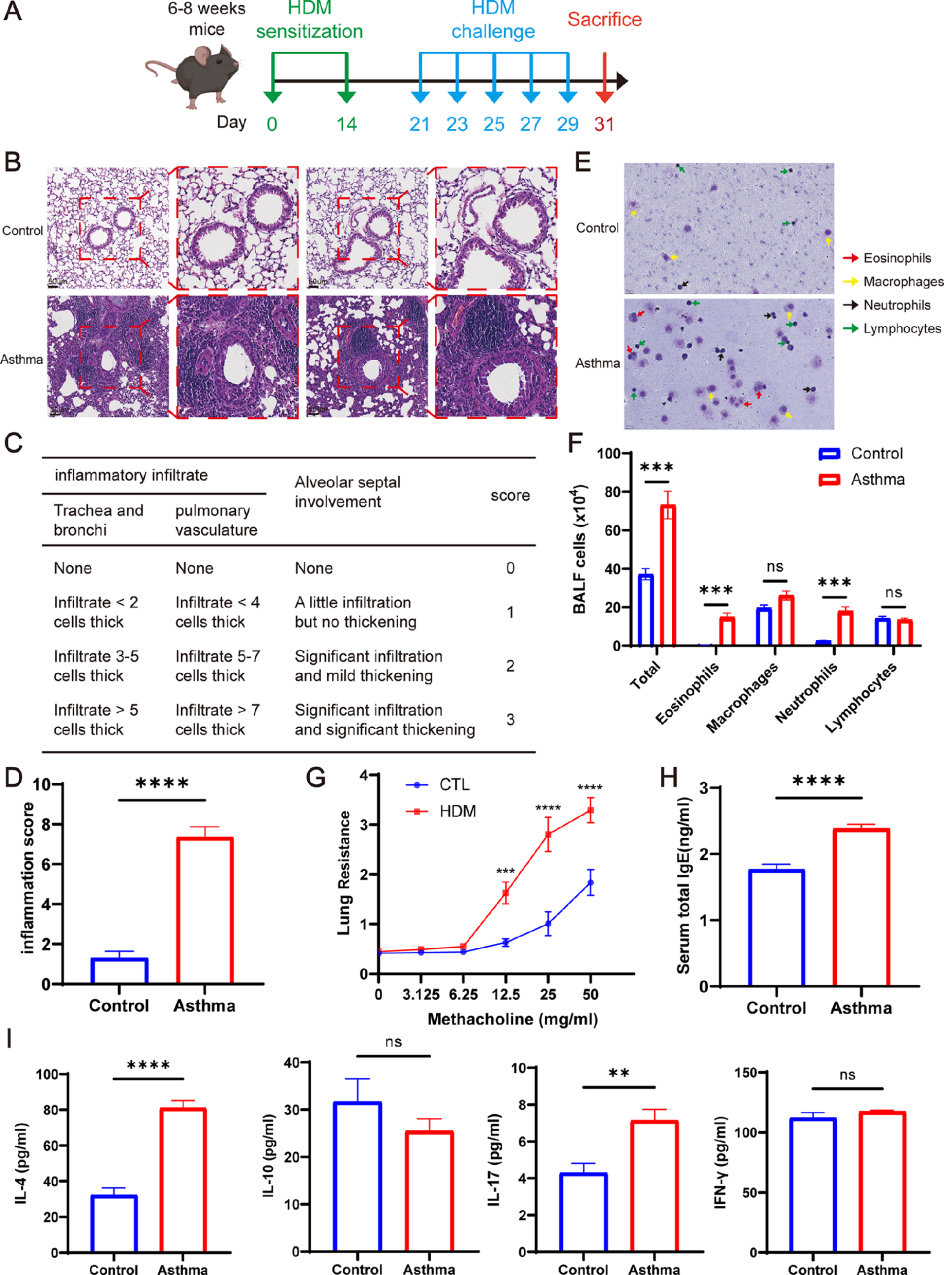
HDM-induced exacerbation of lung inflammation in asthmatic mice. (**A**) Establishment of mice-asthma model induced by HDM. (**B**) Representative lung tissue sections stained with hematoxylin and eosin (H&E). Scale bar = 4 μm. (**C**) Lung tissue inflammation scale in mice. (**D**) The scores of lung inflammation in mice. (**E**) Rachel’s staining of BALF cells. (**F**) Total cell count and leukocyte differential count in BALF. (**G**) Detection of lung resistance in mice after inhalation of methacholine. (**H**) Total serum IgE content of mice. (**I**) Expression of IL-4, IL-10, IL-17 and IFN-γ in BALF supernatant of mice. *P < 0.05; **P < 0.01; ***P < 0.001 ****P < 0.0001

To establish a mouse model of asthma, we sensitized and challenged C57BL/6 mice aged 6–8 weeks with HDM (Fig. 3A). Subsequently, on the 31st day of modeling, the mice were euthanized, and relevant experimental tests and analyses were conducted to validate the successful establishment of the mouse asthma model. The histological analysis of mouse lung tissues using HE staining revealed a marked increase in inflammatory cell infiltration around the trachea, bronchioles, and blood vessels in the asthma group as compared to the control group. Additionally, the lung interstitium exhibited significant thickening (Fig. 3B). According to the pathological findings, we conducted a scoring of lung inflammation (Fig. 3C). The results demonstrated a notably higher inflammation score in the lungs of the mice in the asthmatic group compared to those in the control group (Fig. 3D, *P* < 0.0001). Compared to the control group, mice in the asthma group showed a significant increase in total cell count, eosinophil count, and neutrophil count in the BALF (Fig. 3E-F, *P* < 0.001). Furthermore, non-invasive pulmonary function testing indicated that the lung resistance of both groups of mice increased gradually with the rise of acetyl-β-methylcholine concentration. Once the concentration of acetyl-β-methylcholine reached 12.5 mg/ml, 25 mg/ml, and 50 mg/ml, the lung resistance of the mice in asthma group significantly surpassed that of the mice in control group (Fig. 3G, *P* < 0.001).

In addition, the total IgE levels in the serum were determined, revealing significantly higher levels in the asthma group than in the control group (Fig. 3H, *P* < 0.0001). ELISA was utilized to measure the levels of IL-4, IL-10, IL-17, and IFN-γ in the BALF of the mice. The findings revealed that the levels of IL-4 and IL-17, as pro-inflammatory cytokines in the BALF of the asthmatic group mice, were significantly higher than those in the control group (Fig. 3I, P *<* 0.01). Additionally, no significant variance was observed in the expression levels of IL-10 and IFN-γ between the asthma group and the control group (Fig. 3I). These findings indicate the successful establishment of a mouse asthma model induced by HDM.

### TFEB expression is increased in the lungs of mice with HDM-induced asthma

**Fig. 4:**
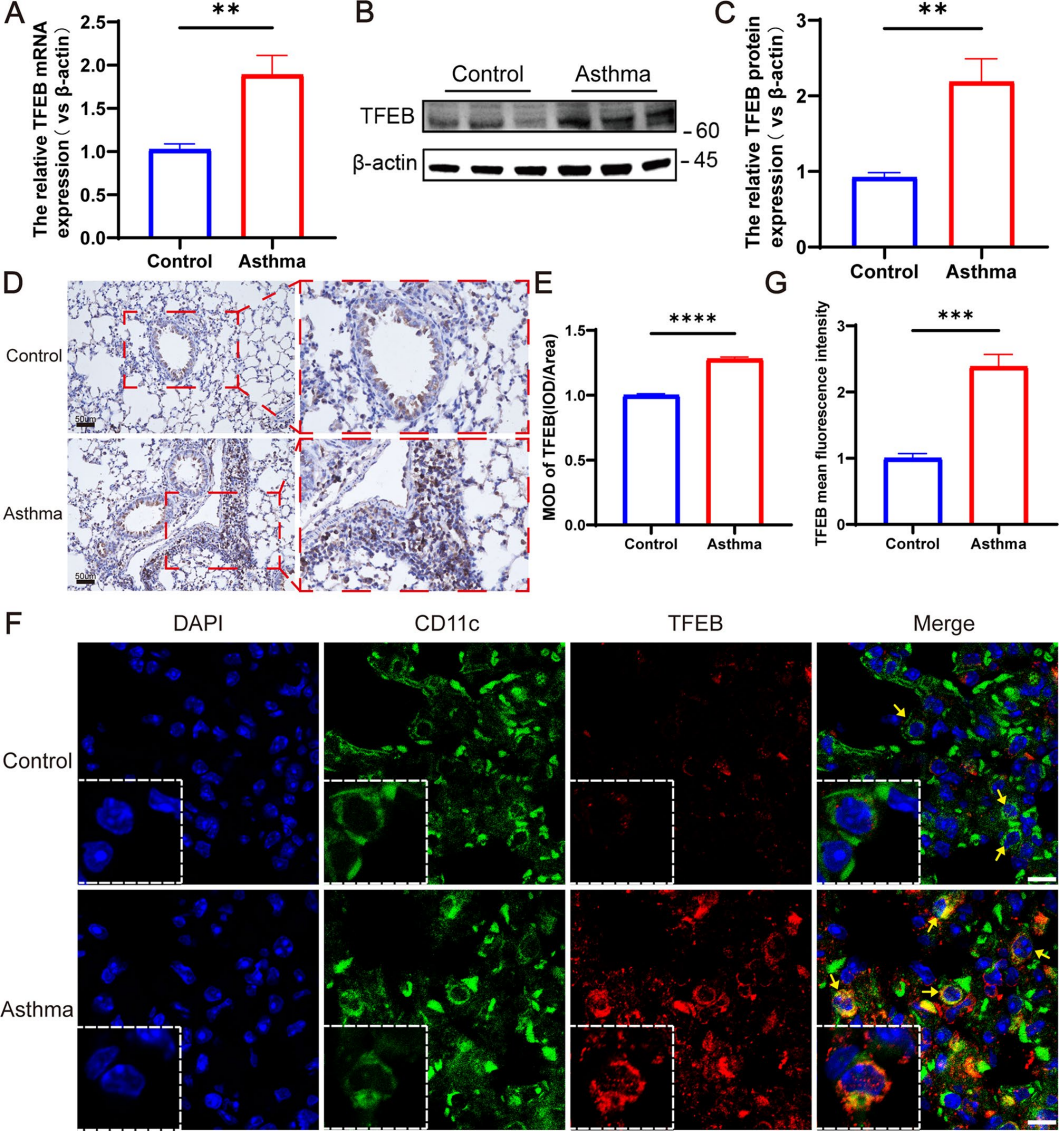
TFEB expression is increased in lung tissues of HDM-induced asthma mice. (**A**) TFEB mRNA expression in lung tissues of HDM-induced asthma mice and control mice. (**B**) Expression and quantification of TFEB protein in lung tissues via western blotting. (**C**) Quantification of western blotting results. (**D**) Immunohistochemical staining of TFEB in lung tissue of mice. (**E**) Quantification of immunohistochemical staining. (**F**) Immunofluorescent staining was performed on lung dendritic cells from mouse lung sections. In the images, blue fluorescence indicates nuclear staining, green fluorescence represents CD11c + dendritic cells, and red fluorescence represents TFEB. (**G**) Mean fluorescence intensity of TFEB in lung tissue immunofluorescence. **P < 0.01; ***P < 0.001 ****P < 0.0001

To detect TFEB mRNA expression in mouse lungs, RT-qPCR was employed. The results revealed a significantly elevated TFEB mRNA level in the lung tissue of mice within the asthma group compared to the control group (Fig. 4A, *P* < 0.01). The Western blot analysis revealed a significant increase in TFEB protein expression in the lung tissue of mice in the asthma group compared to the control group (Fig. 4B-C, *P* < 0.01). Immunohistochemistry results showed that the lungs of mice in the asthma group had a large number of inflammatory cells infiltrated and TFEB was highly expressed (Fig. 4D-E, *P* < 0.0001). Additionally, immunofluorescence of sections showed significantly higher TFEB expression on DCs in the asthma group compared to the control group (Fig. 4F-G, *P* < 0.001). The above results collectively demonstrated an increase in TFEB expression in DCs within the lung tissues of HDM-induced asthmatic mice, corroborating our previous sequencing findings.

### TFEB expression increased in mouse BMDCs after HDM stimulation

**Fig. 5:**
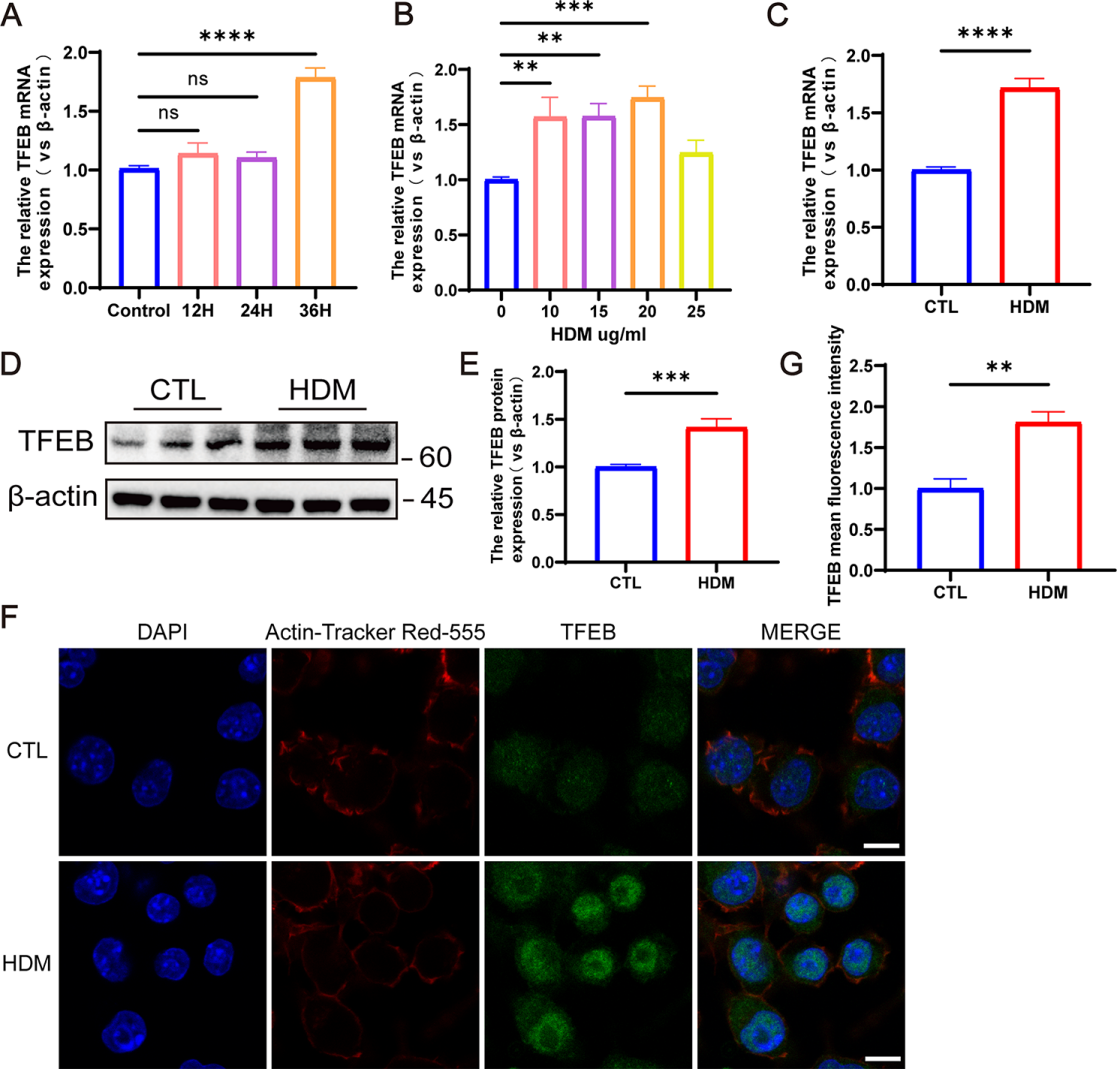
TFEB expression is increased after HDM stimulation of mouse BMDCs. (**A**) TFEB expression in BMDCs stimulated by different concentrations of HDM. (**B**) TFEB expression in BMDCs after HDM stimulation at different times. (**C**) TFEB mRNA expression in BMDCs after 36 h stimulation with HDM at 20 ug/ml. (**D**) TFEB protein expression in BMDCs after 36 h of HDM stimulation at 20 ug/ml. (**E**) Quantification of western blotting results. (**F**) Immunofluorescence staining of TFEB in BMDCs following 36-hour stimulation with 20 µg/ml HDM. Blue fluorescence represents DAPI, red fluorescence represents Actin-Tracker Red-555, and green fluorescence represents TFEB. (**G**) Quantification of the mean fluorescence intensity of TFEB. **P < 0.01; ***P < 0.001; ****P < 0.0001

BMDCs were stimulated with HDM concentrations ranging from 0 to 25 µg/ml for 36 h. The results revealed a concentration-dependent increase in TFEB expression corresponding to the HDM concentration, with the most significant and statistically significant increase observed at 20 µg/ml (Fig. 5A, *P* < 0.001). Subsequently, BMDCs were stimulated with a concentration of 20 µg/ml HDM for 36 h, leading to elevated TFEB expression levels compared to the control group, with a statistically significant difference observed at 36 h of stimulation (Fig. 5B, *P* < 0.0001). Consequently, we selected to stimulate mouse BMDCs with 20 µg/ml of HDM for 36 h for subsequent experiments. RT-qPCR (Fig. 5C), western blot (Fig. 5D-E), and immunofluorescence (Fig. 5F-G) results demonstrated an elevation in TFEB expression following HDM stimulation of BMDCs (*P* < 0.01), with enhanced nuclear expression.

### TFEB expression is inhibited after transfection of BMDCs with lentivirus

**Fig. 6:**
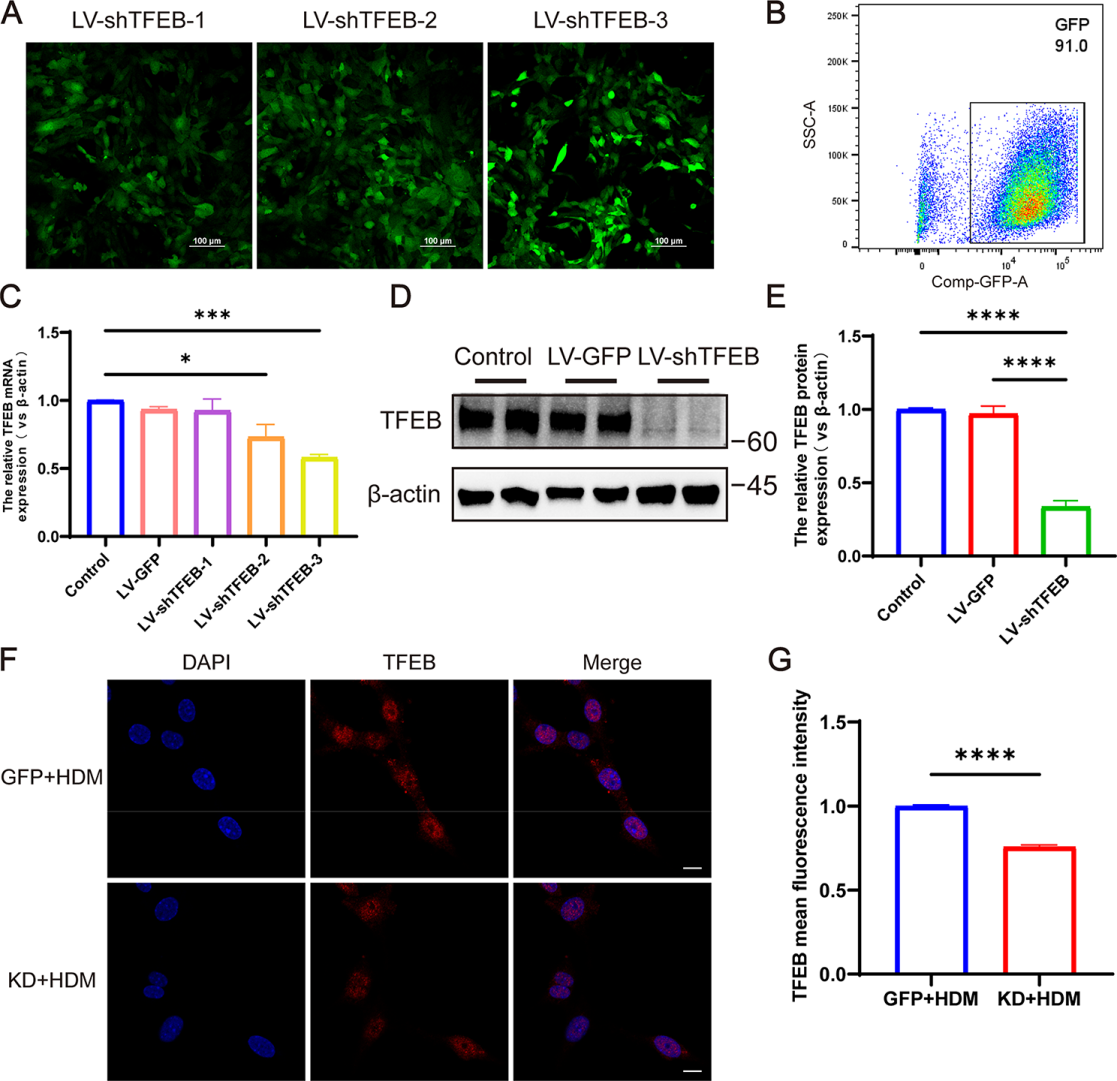
TFEB expression decreased after transfection of BMDCs with lentivirus. (**A**) Confocal microscopy of three lentivirally transfected BMDCs. (**B**) The transfection efficiency of cells by flow cytometry. (**C**) The expression of TFEB mRNA in BMDCs after transfection with 3 different lentiviruses. (**D**) Western blots analysis of TFEB from these three groups. (**E**) Quantification of western blotting results. (**F**) Immunofluorescent staining for TFEB in HDM-treated LV-GFP and LV-shTFEB BMDCs. (**G**) Quantification of the mean fluorescence intensity of TFEB. *P < 0.05; ***P < 0.001 ****P < 0.0001

To investigate the role of TFEB in DCs, BMDCs were transfected with LV-shTFEB, and lentiviral transfection was visualized using confocal microscopy (Fig. 6A). Flow cytometry analysis revealed that approximately 90% of cells were GFP-positive post-transfection (Fig. 6B). Subsequent RT-qPCR analysis showed that LV-shTFEB-3 achieved the most effective knockdown, resulting in a significant reduction of approximately 50% in TFEB expression (Fig. 6C, *P* < 0.001). Consequently, LV-shTFEB-3 was selected for further experiments. Western blot results confirmed the successful knockdown of TFEB (Fig. 6D-E, *P* < 0.0001). Immunofluorescence analysis further demonstrated a reduction in TFEB expression following lentiviral transfection of BMDCs (Fig. 6F-G, *P* < 0.0001). These findings collectively indicate that LV-shTFEB effectively reduces TFEB expression after BMDCs transfection.

### Knockdown of TFEB inhibits the migratory capacity of BMDCs

**Fig. 7:**
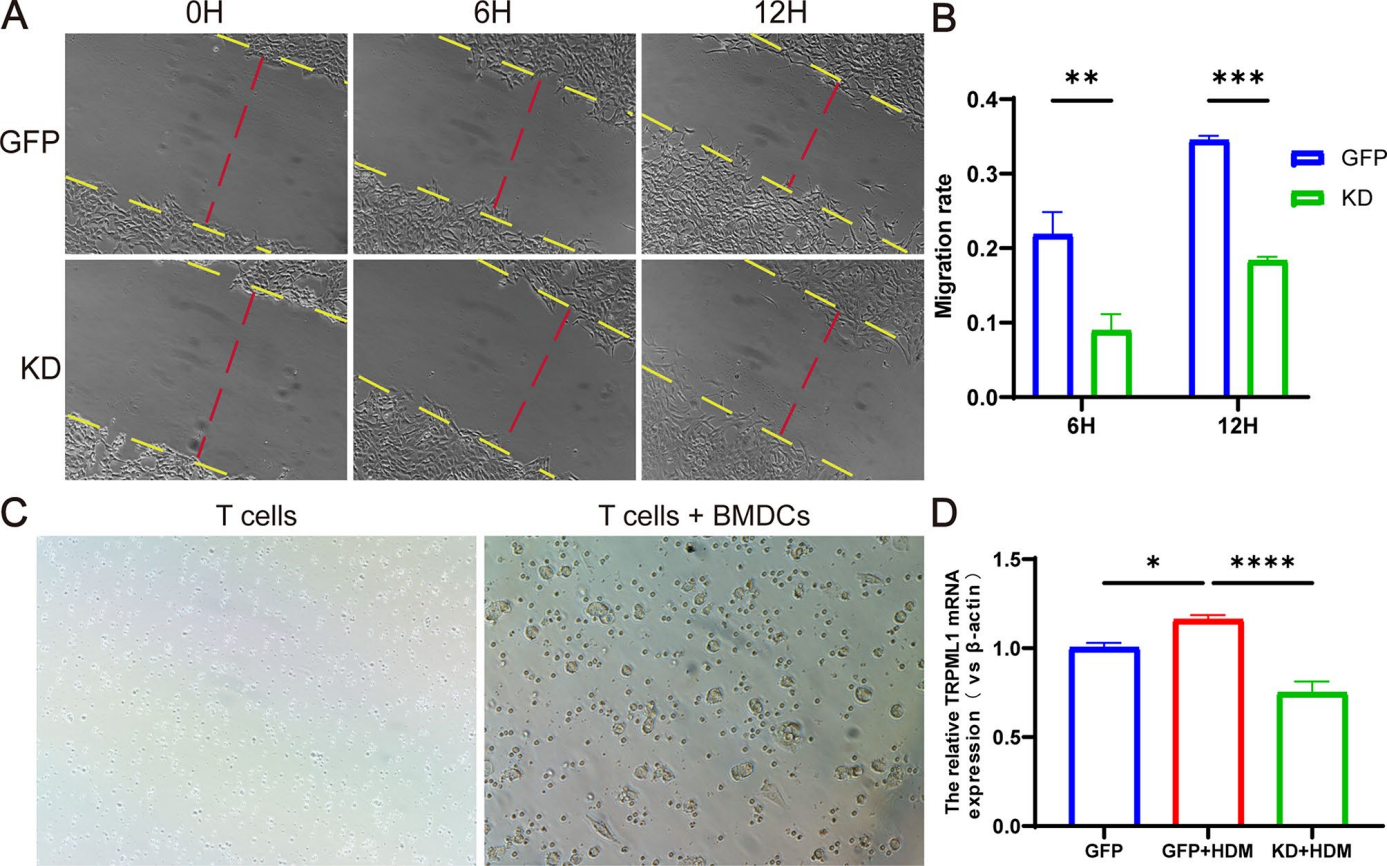
Knockdown of TFEB inhibits the migratory capacity of BMDCs. (**A**) The migratory capacity of BMDCs determined by wound healing assays. (**B**) Quantification of Wound-Healing Assay results. (**C**) BMDCs were co-cultured with spleen T cells. (**D**) The expression of TRPML1 mRNA in co-cultured cells detected by RT-qPCR. *P < 0.05; **P < 0.01; ***P < 0.001; ****P < 0.0001

The migration ability can reflect the antigen presentation and activation status of BMDCs. Therefore, we first investigated whether knocking down TFEB affects the migration ability of BMDCs. The cell wound healing assay results demonstrated a significant inhibition of BMDCs migration following TFEB knockdown (Fig. 7A-B, *P* < 0.01). Next we co-cultured BMDCs knocked down TFEB with spleen T cells (Fig. 7C). As previously mentioned, the migration of DCs to lymph nodes may be positively regulated by the TFEB-TRPML1 axis, so we examined the expression levels of TRPML1 molecules in the co-cultured cells by RT-qPCR. We found that the expression of TRPML1 molecules was elevated after HDM stimulation of the co-cultured cells (Fig. 7D, *P* < 0.05), and the expression of TRPML1 molecules was significantly decreased after knockdown of TFEB (Fig. 7D, *P* < 0.0001).

### Knockdown of TFEB inhibits BMDCs’ antigen presentation capability

**Fig. 8:**
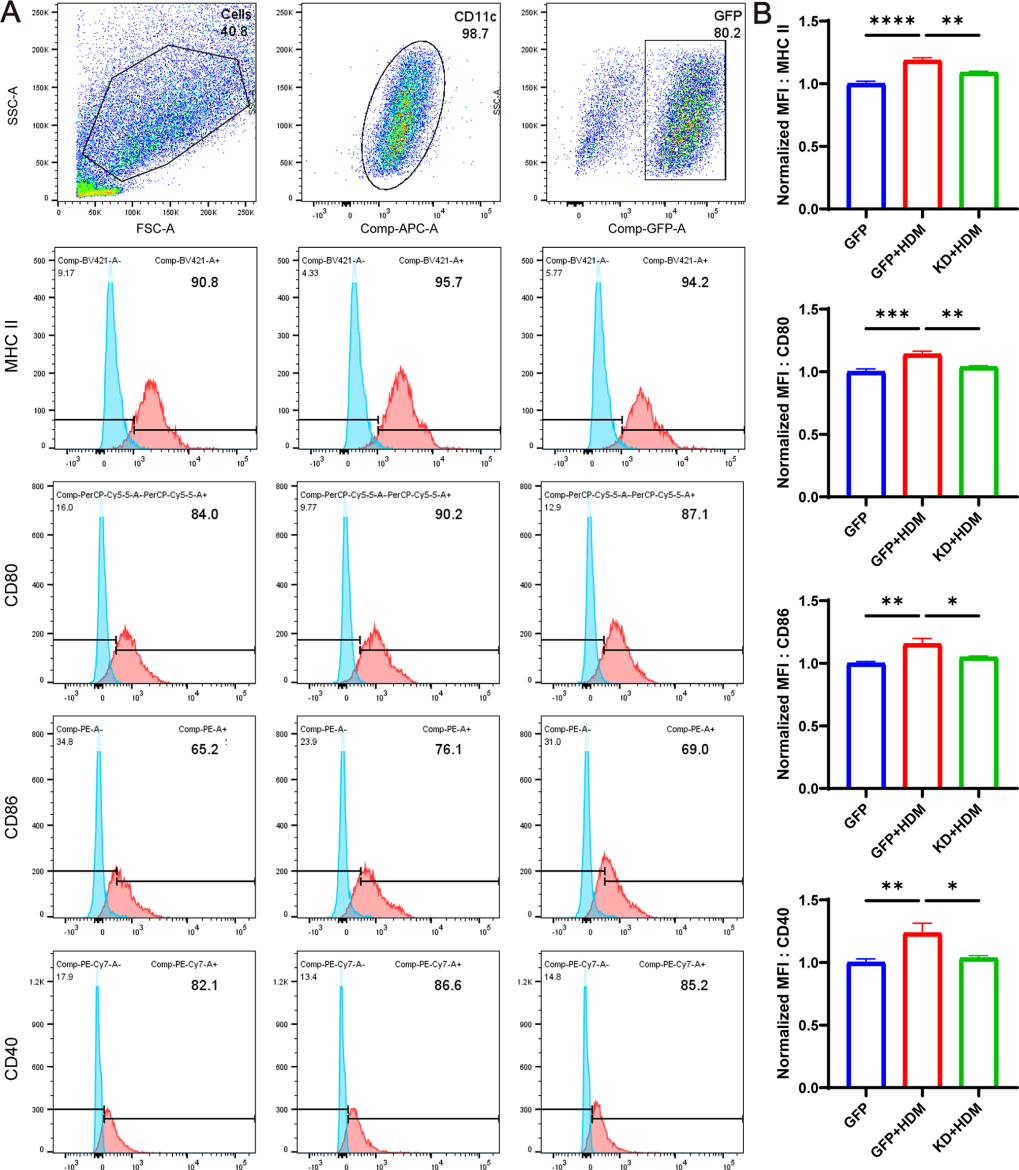
Knockdown of TFEB inhibits the antigen presentation ability of BMDCs. (**A**) The expression of MHC II, CD80, CD86 and CD40 molecules on the surface of BMDCs was analyzed by flow cytometry. (**B**) Statistical analysis of flow cytometry. *P < 0.05; **P < 0.01; ***P < 0.001; ****P < 0.0001

In order to investigate whether knocking down TFEB affects the antigen presentation process of BMDCs, we first used flow cytometry to detect the expression of antigen recognition molecule MHCII and co-stimulatory molecules CD40, CD80, and CD86 on the surface of BMDCs in co-cultured cells to assess the antigen presentation ability of BMDCs. The results revealed that following HDM stimulation, the Mean Fluorescence Intensity (MFI) of surface molecules MHCII, CD40, CD80, and CD86 on BMDCs increased. However, upon knockdown of TFEB, these MFIs decreased significantly (Fig. 8A-B, *P* < 0.05).

### Knockdown of TFEB in BMDCs co-cultured with T cells can reshape the balance of Th1/Th2 and Treg/Th17 cells

**Fig. 9:**
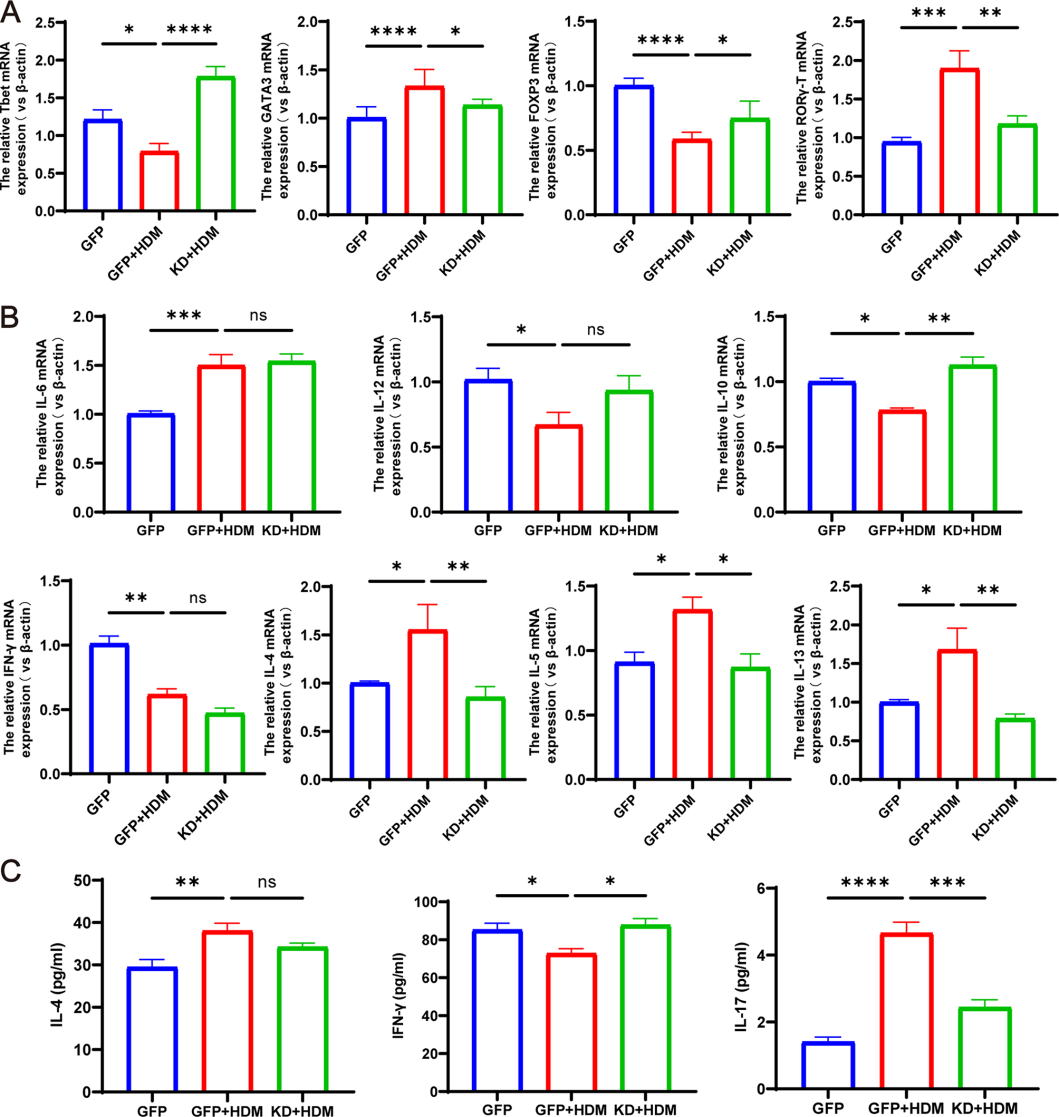
Co-culture of TFEB-knockdown BMDCs with T cells remodels the balance of Th1/Th2 and Treg/Th17 cells. (**A**) T- bet, GATA3, Foxp3 and RORγt mRNA expression in each group. (**B**) The expression of IL-6, IL-12, IL-10, IFN-γ, IL-4, IL-5 and IL-13 in co-cultured cells detected by RT-qPCR. (**C**) Expression of IL-4, IFN-γ and IL-17 in the supernatant of co-cultured cells detected by ELISA. *P < 0.05; **P < 0.01; ***P < 0.001; ****P < 0.0001

In order to further investigate the impact of BMDCs on T cell subpopulation differentiation after knockdown of TFEB, we examined the expression levels of factors such as FOXP3, RORγ-T, GATA3 and T-bet in co-cultured cells by RT-qPCR. Compared with the control group, the expression of FOXP3 and T-bet in co-cultured cells after HDM stimulation was significantly decreased, while the expression of RORγ-T and GATA3 was significantly increased. However, knockdown of TFEB resulted in increased expression of FOXP3 and T-bet and decreased expression of RORγ-T and GATA3 in co-cultured cells (Fig. 9A, *P* < 0.05). To examine the impact of TFEB knockdown on cytokine secretion by BMDCs and T cells, we used RT-qPCR to detect the expression levels of cytokine mRNA such as IL-6, IL-10, IL-12, IFN-γ, IL-4, IL-5, and IL-13 in co-cultured cells. Additionally, we measured the expression of IL-4, IFN-γ and IL-17 in the supernatants of co-cultured cell medium through ELISA. After HDM stimulation, we observed a decrease in IFN-γ secretion by T cells, an increase in the expression of IL-4, IL-5, IL-13, and an elevation in IL-17 secretion. These alterations were mitigated following TFEB knockdown (Fig. 9B-C, *P* < 0.05). It indicates that knocking down TFEB alters the expression or secretion of pro-inflammatory/anti-inflammatory cytokines, which may be related to its reshaping of the balance of Th1/Th2 and Treg/Th17 cells.

## Discussion

**Fig. 10:**
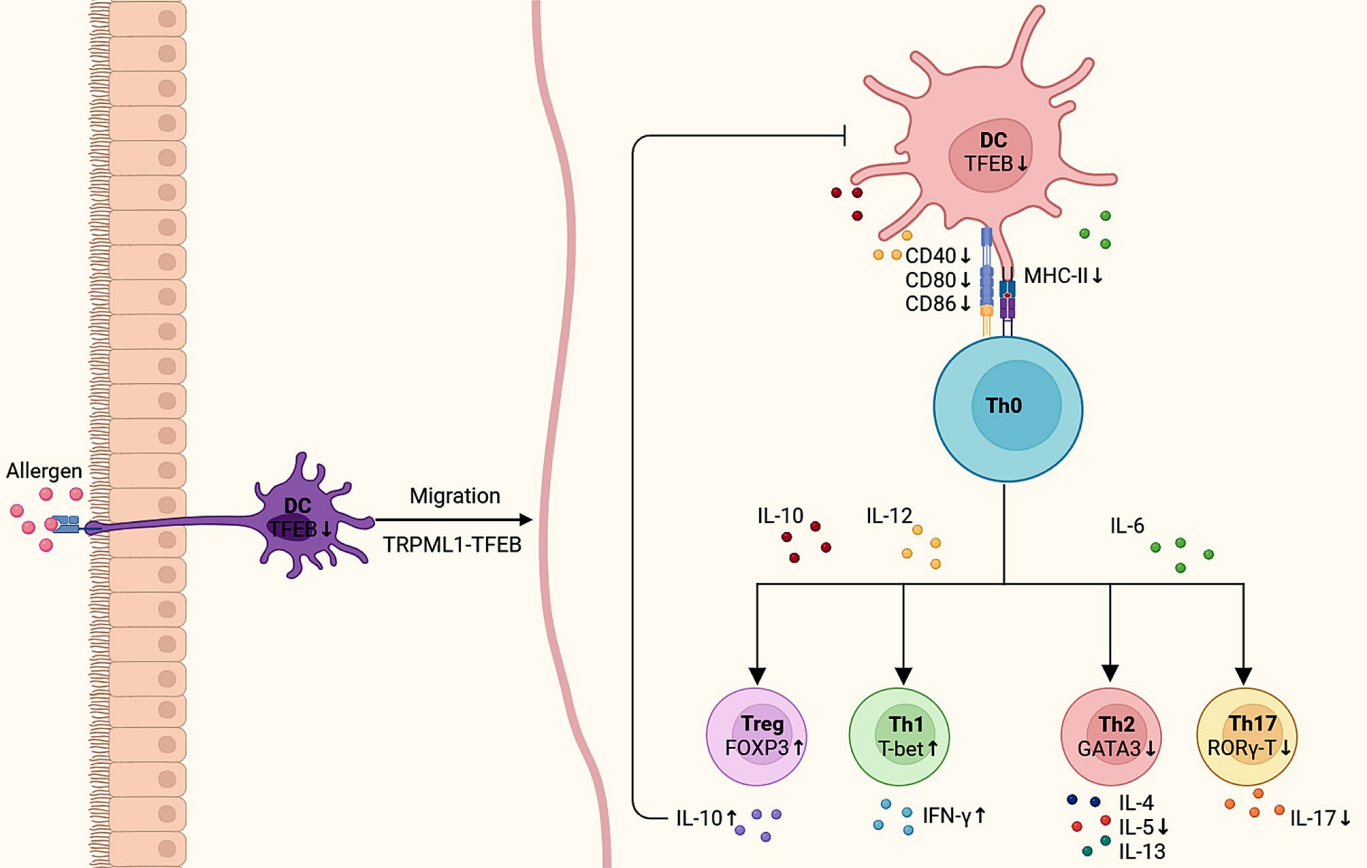
Schematic representation of knockdown of TFEB regulating Th0 cell subpopulation differentiation through inhibition of dendritic cell antigen presentation. The suppression of TFEB hindered the migratory ability of dendritic cells and reduced the surface expression of CD40, CD80, CD86, and MHC-II on DC cells, thereby impacting the differentiation of Th0 cell subtypes. This inhibition led to decreased expression of pro-inflammatory factors and increased expression of anti-inflammatory factors, ultimately rebalancing the Th1/Th2 and Treg/Th17 cell equilibrium

In this study, our initial RNA-seq analysis validated the differential expression of the TFEB gene in HDM-induced BMDCs, suggesting its potential involvement in immune system modulation and antigen presentation pathways. Next, we confirmed the increased expression of TFEB in PBMCs from asthmatic children as well as in the lung tissues of asthmatic mice. By constructing a co-culture system of BMDCs and T cells, we observed that knocking down TFEB inhibited the migration and surface expression of antigen presentation and co-stimulatory molecules of BMDCs. The above results indicate that the knockdown of TFEB inhibited the antigen presentation process of DCs. Furthermore, we observed that the knockdown of TFEB affected the differentiation of naïve T cells, resulting in a decrease in the expression or secretion of pro-inflammatory cytokines and an increase in the expression or secretion of anti-inflammatory cytokines. This led to a re-regulation of the balance between Th1/Th2 and Treg/Th17 cells (Fig. 10).

The pathogenesis of asthma is very complex, and it is currently believed to be mainly related to the antigen presentation of dendritic cells and T cell activation [19, 20]. Dendritic cells are considered to be the most important antigen-presenting cells, mediating immune responses and tolerance of T cells by continuously presenting co-stimulatory molecules and/or cytokines to T cells [21]. The activation of T cells requires the completion of a dual-signal pathway involving DCs, which includes the antigen recognition signal and the co-stimulatory signal. The antigen recognition signal acts as the first signal, during which dendritic cells present exogenously derived peptide antigens to CD8 + T cells and CD4 + T cells by expressing MHC class I and MHC class II molecules. The co-stimulatory molecules on the surface of dendritic cells then bind to and interact with the corresponding receptors on the surface of T cells to initiate a second signal, the coordinated stimulatory signal [22–24]. In asthma, dendritic cells present antigens to CD4 + T cells by expressing MHC class II molecules and rely on co-stimulatory molecules such as CD40, CD80, CD86 to induce T cell activation [20, 25]. In addition, the cytokines secreted by DCs after antigen stimulation can also regulate the subset differentiation of naïve T helper cells [26, 27]. Among them, IL-12 and IFN-γ are important cytokines in inducing the differentiation of Th0 cells into Th1 cells [28]. IL-6 induces Th0 to Th2 and/or Th17 differentiation [29]. Whereas IL-10 induces the differentiation of Th0 cells into Treg cells. At the same time, IL-10 can also act on DCs to inhibit their maturation and suppress the expression of MHC-II, co-stimulatory molecules, and chemokines [27, 30, 31]. Therefore, after clarifying the antigen presentation of DCs and the immune response process of T cells in asthma, we want to identify important regulatory targets for this process from the source, in order to provide more effective prevention and treatment for asthma.

TFEB is one of the MiTF/TFE family members of the leucine zip (bHLH-LZ) class of transcription factors, which have been found to potentially play an important role in a variety of diseases such as lysosomal storage disorders, neurodegenerative diseases, hepatic metabolic disorders, obesity, and others [9, 10, 32]. Liu et al. found that promotion of nuclear translocation in tumour cell TFEB was able to induce PD-L1 degradation and mediate anti-tumour immune responses [33]. In another study, TFEB was also shown to synergistically regulate the involvement of B cells in the humoral immune response by regulating CD40L expression in T cells [11]. Furthermore, in a mouse model of autoimmune disease, deletion of TFEB in Treg cells resulted in reduced Treg accumulation and impaired Treg function [34]. The above studies suggest that TFEB may play an important role in regulating immune cell-mediated immune responses. Based on the above research background, we want to understand whether TFEB is involved in the immune response of asthma DCs. Our study results first suggested that TFEB has differential expression in asthma DCs, and may be involved in antigen presentation and the immune response process. The above results lay the groundwork for further exploration of the role of TFEB in antigen presentation by DCs. Recent studies have identified TFEB as a key transcription factor regulating lysosomes, and the migration of DCs from the periphery to the lymph nodes is controlled by a positive feedback loop mediated by the TFEB-TRPML1 axis in lysosomes [16, 35]. In this study, we also observed that after knocking down TFEB, the migration of BMDCs was significantly inhibited, and the expression of TRPML1 in BMDCs decreased. At the same time, our research results also suggest that knocking down TFEB will inhibit the expression of MHC II molecules, as well as co-stimulatory molecules CD80, CD86, and CD40 on the surface of BMDCs, indicating that TFEB may be involved simultaneously in antigen recognition and co-stimulation of DCs, affecting the antigen presentation process of DCs.

It is currently believed that the imbalance of Th1/Th2 and/or Th17/Treg cell subgroups and the excessive secretion of cytokines are critical factors in the pathogenesis of allergic asthma [36–39]. Therefore, we further investigated whether regulating the expression of TFEB affects the differentiation of Th0 cell subgroups and the expression of cytokines. Initially, we investigated the mRNA expression of T-bet, GATA3, RORγ-T, and Foxp3 in co-culture systems of BMDCs and T cells induced by HDM. These transcription factors play a crucial role in regulating the differentiation of Th0 cells into Th1, Th2, Th17, and Treg cells, respectively, and thus were utilized to signify the differentiation of subpopulations within the Th0 cells [40–42]. Our results indicate that the expression of GATA3 and RORγ-T decreased after knocking down TFEB, while the expression of T-bet and FOXP3 increased. This suggests that the differentiation of Th0 towards Th2 and Th17 is inhibited after knocking down TFEB, while the differentiation towards Th1 and Treg is increased. Therefore, knocking down TFEB can rebalance the Th1/Th2 and Treg/Th17 cells. In addition, the differentiation of Th0 cells may also be regulated by the cytokines secreted by DCs [27]. However, our research results show that the knockdown of TFEB did not significantly alter the cytokines (IL-6 and IL-12) secreted by DCs, indicating that TFEB does not completely depend ons this pathway but is more likely to affect the differentiation of T cells by inhibiting the antigen presentation process of DCs.

It is well known that IFN-γ, IL-4/IL-5/IL-13 and IL-17 are pro-inflammatory cytokines expressed or secreted by activated Th1, Th2 and TH17 cells, respectively, and play key roles in various immune diseases [42]. In contrast, Treg cells can express or secrete the anti-inflammatory cytokine IL-10, which is thought to have immunosuppressive functions and play an important role in immune tolerance [43, 44]. In this study, we found that knockdown of TFEB inhibited the expression of IL-4, IL-5, and IL-13, while inhibiting the secretion of IL-17, and increasing the secretion of IFN-γ and the expression of IL-10. The aforementioned findings suggest that upon TFEB knockdown in DCs, there is a decrease in pro-inflammatory factors and an increase in anti-inflammatory factors, providing additional evidence that modulating TFEB can recalibrate the Th1/Th2 and Treg/Th17 cell balance.

## Conclusion

In summary, our study demonstrated up-regulation of TFEB expression in asthmatic dendritic cells. Manipulating TFEB expression enabled regulation of dendritic cell migration and antigen presentation processes, modulation of the immune response of CD4 + T cells, and remodeling of the subpopulation balance of Th1/Th2 and Th17/Treg cells. These findings are anticipated to identify new therapeutic targets for preventing and treating asthma, with potential clinical applications.

### Electronic supplementary material

Below is the link to the electronic supplementary material.

Supplementary Material 1

## Data Availability

All data generated or analysed during this study are included in this published article. The datasets used and/or analysed during the current study are available from the corresponding author on reasonable request. Data obtained from RNA-seq, which provide the evidence for the conclusions made in this study, are supervised by FengXia Ding. Access to these data is restricted, as they were generated under a license specific to the current study and are not publicly available. Nonetheless, the author can make the data available upon reasonable request and with FengXia Ding’s permission. FengXia Ding’spermission.

## Abbreviations

DCs: Dendritic cells
TFEB: Transcription Factor EB
HDM: House dust mite
BMDCs: Bone marrow dendritic cells
PBMCs: Peripheral blood mononuclear cells
Treg: Regulatory T cell
DEGs: Differential genes
RNA-seq: RNA sequencing
H&E: Hematoxylin and eosin
AHR: Airway hyperresponsiveness
ELISA: Enzyme-linked immunosorbent assay
IHC: Immunohisto-chemical
MOD: Mean optical density
BALF: Bronchoalveolar lavage fluid
LV: Lentiviral vectors
GFP: Green fluorescent protein
